# Effects of achromatic and chromatic lights on pupillary response, endocrinology, activity, and milk production in dairy cows

**DOI:** 10.1371/journal.pone.0253776

**Published:** 2021-07-22

**Authors:** Sofia Lindkvist, Emma Ternman, Sabine Ferneborg, Daniel Bånkestad, Johan Lindqvist, Björn Ekesten, Sigrid Agenäs

**Affiliations:** 1 Department of Animal Nutrition and Management, Faculty of Veterinary Medicine and Animal Science, Swedish University of Agricultural Sciences, Uppsala, Sweden; 2 Department of Animal and Aquacultural Sciences, Faculty of Biosciences, Norwegian University of Life Sciences, Ås, Norway; 3 Department of Horticulture and Technology, Heliospectra AB, Gothenburg, Sweden; 4 Department of Clinical Sciences, Faculty of Veterinary Medicine and Animal Science, Swedish University of Agricultural Sciences, Uppsala, Sweden; University of Illinois, UNITED STATES

## Abstract

Artificial light can be used as a management tool to increase milk yield in dairy production. However, little is known about how cows respond to the spectral composition of light. The aim of this study was to investigate how dairy cows respond to artificial achromatic and chromatic lights. A tie-stall barn equipped with light-emitting diode (LED) light fixtures was used to create the controlled experimental light environments. Two experiments were conducted, both using dairy cows of Swedish Red and light mixtures with red, blue or white light. In experiment I, the response to light of increasing intensity on pupil size was evaluated in five pregnant non-lactating cows. In experiment II 16h of achromatic and chromatic daylight in combination with dim, achromatic night light, was tested on pregnant lactating cows during five weeks to observe long term effects on milk production, activity and circadian rhythms. Particular focus was given to possible carry over effects of blue light during the day on activity at night since this has been demonstrated in humans. Increasing intensity of white and blue light affected pupil size (P<0.001), but there was no effect on pupil size with increased intensity of red light. Milk yield was maintained throughout experiment II, and plasma melatonin was higher during dim night light than in daylight for all treatments (P<0.001). In conclusion, our results show that LED fixtures emitting red light driving the ipRGCs indirectly via ML-cones, blue light stimulating both S-cones and ipRGCs directly and a mixture of wavelengths (white light) exert similar effects on milk yield and activity in tied-up dairy cows. This suggests that the spectral composition of LED lighting in a barn is secondary to duration and intensity.

## Introduction

In dairy cows, photoperiod can be used as a management tool to increase milk yield and improve working conditions for barn staff. When artificial light is used to extend a natural 8-h day to 16 h of daylight for lactating cows, milk yield [1, 2] and circulating insulin-like growth factor-1 (IGF-1) increase [3]. It is not known whether the type of light is important for the galactopoietic response, but manufacturers of light-emitting diode (LED) fixtures for dairy barns suggest that specific wavelengths are important for the effect on milk yield. Red light is often promoted by the industry as night light, because it is claimed not to affect the cows’ diurnal rhythm.

LEDs reduce consumption of electricity for illumination in dairy barns and require less maintenance compared with several other types of light fixtures available for animal houses, which makes them increasingly popular. The use of LEDs also entails better control of light intensity as the diodes can be dimmed, as well as better control of the light spectrum as there are many different color types available. Artificial light supplements daylight, when daylight is available, and provides adequate levels of illuminance during the rest of the day to allow a daylight-like environment of 16h per 24h for lactating cows [4]. Humans respond differently to natural light compared to artificial light [5], and little is known about how cows respond to lights of different spectral composition. With the increasing use of LED light on dairy farms, it is interesting to investigate whether specific wavelength mixes are beneficial for increased milk production.

Mammals, including cattle have two major types of photoreceptors, cones and rods, that are involved in vision [6]. Cattle, like most mammals, are dichromats and have short-wavelength-sensitive (S-cones) and medium- to long-wavelength-sensitive cones (ML-cones) with opsins peaking at 451 (blue) and 555 (greenish-yellow) nm, respectively [7]. However, the eye also provides sensory input for non-image-forming visual functions, including circadian photo entrainment for setting internal biological clocks, inhibition of melatonin release, which plays a pivotal role in the sleep-wake cycle, and adjustment of the number of photons reaching the retina through the pupillary light reflex [8–11]. A third group of photosensitive receptors in the retina, intrinsically photosensitive retinal ganglion cells (ipRGCs) containing the photopigment melanopsin, drive or contribute to regulation of all these functions [9, 12–15].

In humans, low light exposure during night-time causes acute suppression of melatonin [16]. Studies in humans have shown that the most potent part of the spectrum for providing circadian input for regulation of melatonin secretion is around 446–477 nm [13, 17, 18]. These wavelengths coincide with the absorption peak of the bovine S-cones (451 nm) and are also close to the peak absorption maximum of melanopsin [9, 12, 13]. There is also substantial evidence that exposure to blue light can increase alertness and stimulate cognitive function in humans [19], also after the blue light is turned off [20, 21]. In dairy calves, blue LED light suppressed the expected melatonin increase in the evening when compared to another treatment with yellow LED light [22]. It is therefore possible that using blue LED light during daytime, or during part of the day, could increase the activity of cows at night also when the lights are considerably dimmed or turned off to allow the animals a break from artificial light.

Furthermore, the incident of photon flux onto the retina is adjusted by the pupil size. For a long time, constriction of the pupil in daylight was considered to be driven by retinal cones and chiefly related to the luminance. More recently, it has been postulated that although the photoreceptors play a role in regulation of pupil size at least when there is a transient change in background light, the size of the steady-state pupil is mainly controlled by the ipRGCs [23, 24]. Pupillary dilation is almost completed at one minute in humans and fully completed in 10 minutes at low light levels [25], whereas pupillary constriction is a very rapid process and retinal cone adaptation also seems to be completed in less than 10 minutes [26]. Hence we decided to study the pupil size in cows under different lighting conditions to understand if pupil size and thereby retinal illumination changed when different lighting regimes were used.

The aim of this study was to investigate the effects of different spectral compositions of artificial light on lactating dairy cows. Specific hypotheses were that: i) Pupil response is driven by photon flux and does not differ between different wavelengths; ii) blue light during the day increases the activity of cows at night; and iii) red light does not support diurnal release pattern of melatonin as well as blue or white light.

## Material and methods

The study was conducted at the Swedish Livestock Research Centre, Uppsala, Sweden, and comprised two experiments. All animal handling was approved by the Uppsala Ethics Committee for Animal Research, Uppsala, Sweden (reference no. 5.2.18-11064/16).

**Fig 1:**
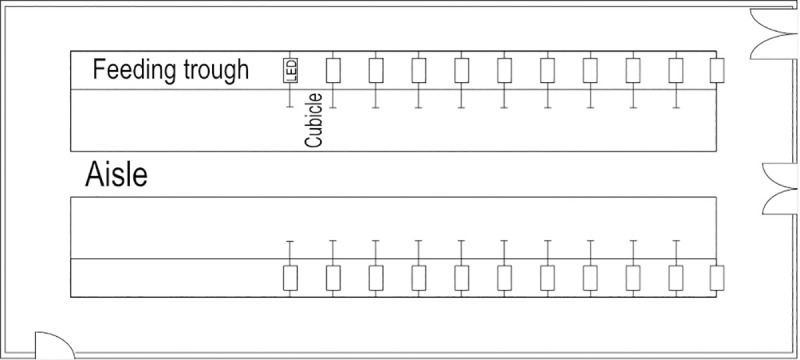
Layout of the Light lab. LED fixtures placed on each side of the head of every cow.

The experiments were performed in a tie-stall barn with a controlled light environment and no contamination from external light (hereafter called the ‘Light lab’). The Light lab had tie-stalls in two rows, on each side of an alley. One light treatment could be applied per row, allowing two treatments to be tested at a time. The tie stalls had rubber mats and wood shavings as bedding material. The stalls were cleaned and bedding material replaced during milking. Water was provided *ad libitum*, from individual automatic water bowls. The Light lab was equipped with LED light fixtures (Elixia LX602G, Heliospectra AB, Sweden) placed on each side of the head of every cow, approximately 140 cm above the forehead (Fig 1). The LEDs in the light fixtures were remotely controlled and hence, both intensity and the spectral composition of the light could be adjusted.

### Light measurements

**Fig 2:**
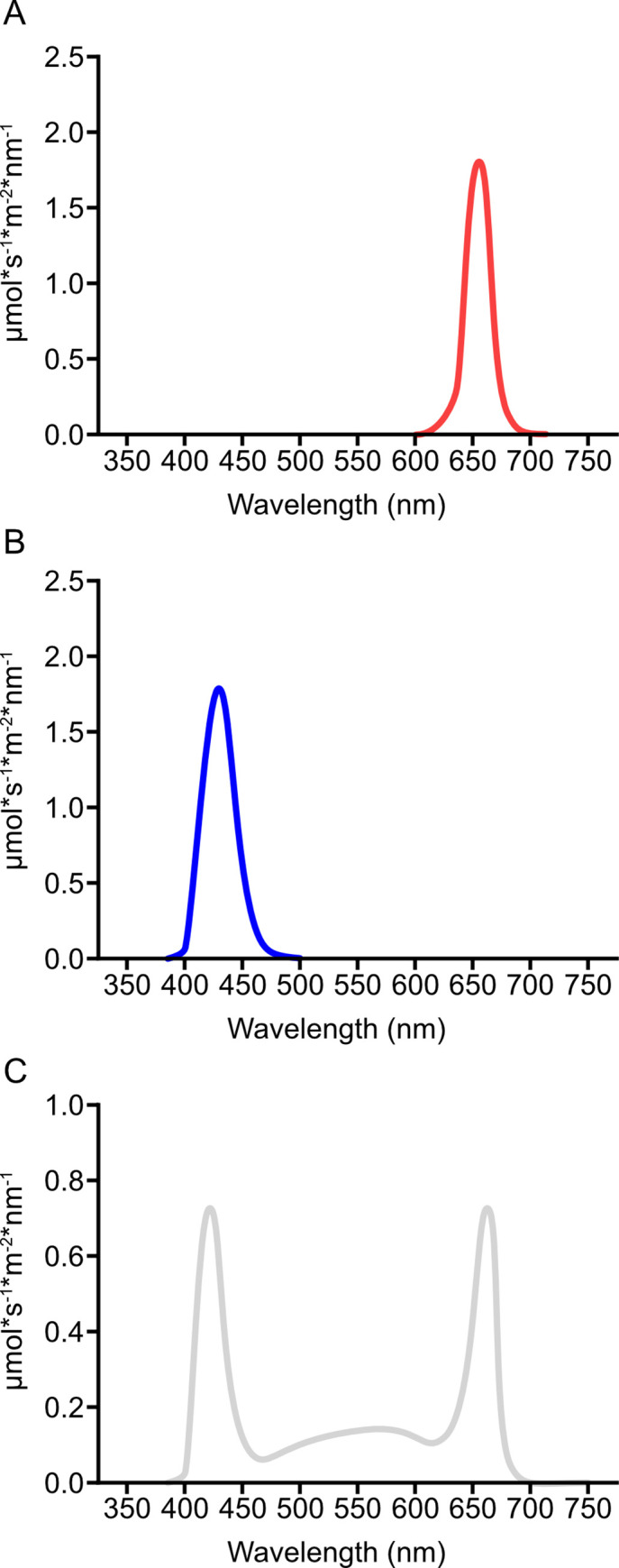
Spectral composition of the light used during daytime in the Light lab. (A) Red light treatments, (B) Blue light treatments, and (C) White light treatments.

Light was measured at the level of the cow eye, approximately 125 cm above the floor, with a photosensor directed towards the ceiling. A luxmeter [Hagner Screenmaster, B. Hagner AB, Solna, Sweden], a photometer [(IL-1700, International Lights, Peabody, MA, USA], and a spectrometer [Jaz, Ocean Insight, Inc. Dunedin, Florida, USA] were used for this purpose, and hence illuminance (lux), luminance (cd/m^2^), photon flux density (μmol s^-1^ m^-2^), and light spectrum (μmol s^-1^ m^-2^ nm^-1^) were quantified. To simplify reporting, we frequently use the expression ‘light intensity’ rather than these four physically correct terms when referring to amount of light in the barn, and we refer to the different mixtures of wavelengths used in the experiments as ‘colors’ based on the hues a normal human trichromat would perceive on seeing the light (Table 1 and Fig 2). The different intensity levels tested (1–10, Table 1) were designed to provide similar photon flux density, while the illuminance and luminance values were used for comparison.

**Table 1:** Light intensity levels used in the experiments, expressed as photon flux density (μmol s^-1^ m^-2^), illuminance (lux), and luminance (candela/m^2^).

Light	Red			Blue			White		
Intensity	Photon flux density	Illuminance	Luminance	Photon flux density	Illuminance	Luminance	Photon flux density	Illuminance	Luminance
1	-	-	-	0.18	1.7	2.2	0.18	11.7	1.87
2	0.36	6.1	0.005	0.4	1.8	4.8	0.4	32	4.8
3	0.83	14.8	0.06	0.73	2.4	12.1	0.62	49.1	9.3
4	1.46	21.4	0.12	1.4	47	24	1.48	115.6	23.7
5	2.78	40.6	0.19	2.85	94	42.1	2.83	219	42.9
6	5.89	86.6	0.49	5.82	127	75.5	5.82	390	85.9
7	11.3	167.4	0.83	11.4	370	158.5	11.6	662	135.7
8	23.4	342	1.72	23.0	773	324	23.1	1070	216
9	34.9	676	31.8	34.7	1674	416	36.9	1668	325
10	-	-	-	-	-	-	46	3550	745

### Experiment I

**Fig 3:**
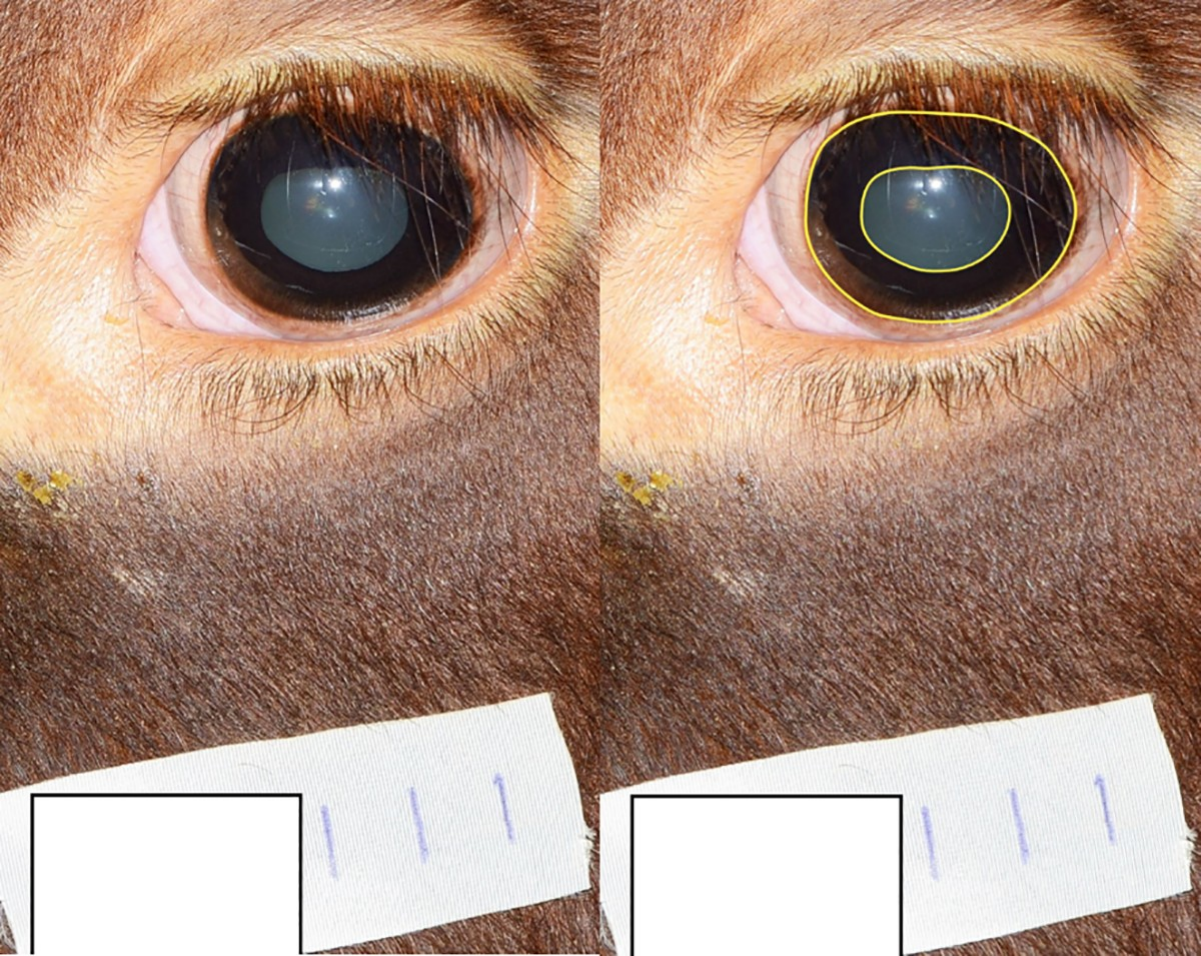
Photograph illustration for the calculation of relative area of the pupil (RAP). The yellow marks in the right photograph indicate the pupil area and the iris area. RAP was calculated by dividing the area covered by the pupil with the area area circumscribed by the peripheral iris at the limbus cornea.

Size of pupils in response to blue, red, and white light of increasing intensity was studied in five pregnant non-lactating cows of the Swedish Red (SRB). The exposure started with the dimmest light (Blue_1_, followed by White_1_, and then Red_2_, Blue_2_, White_2_) and the light intensity was increased step-wise as shown in Table 1. When the cows had adapted to the test light for 10 minutes, photographs of each eye were taken at each light intensity at approximately 2–3 m distance, using a digital camera (Nikon D800 with a Nikon AF-S Nikkor 70-200mm f/2,8 lens). Relative area of the pupil (RAP) was calculated as the area covered by the pupil in the photograph divided by the area circumscribed by the peripheral iris at the *limbus cornea* (Fig 3). To ensure a comparable scale in the photographs, a piece of white surgical tape with a centimeter scale was placed below the eye on every cow. All photographs were analyzed by the same researcher (author S.L.) using imaging software (Adobe Photoshop 2020 version 21.0.3). Cow identity and lighting conditions for each image were blinded for measurements. To estimate the amount of light actually reaching the retina and to enhance comparison with conventional retinal illumination measured in Trolands (which is equal to the pupil area in mm^2^ times the luminance in candela/m^2^) [27], the photon flux was multiplied by mean RAP.

### Experiment II

Forty lactating SRB cows were blocked according to days in milk (range: 117–331), days in pregnancy (range: 31–137), parity (range: 2–7), and daily milk yield (range: 22–45 kg) and randomly assigned to one of two light treatments in each of two periods: Blue (n = 10) and Red (n = 10) in period 1, and White (n = 10) and White-Blue (n = 10) in period 2. Period 1 ran from January to March 2019, and period 2 from March to May 2019.

**Fig 4:**
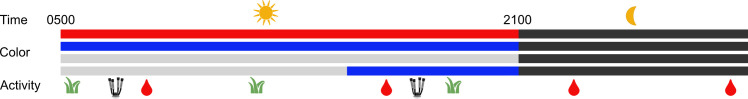
Schematic illustration of the light treatments during 24 hours. Daylight was provided during 16 hours and dim night light during eight hours. Symbols indicate times of feeding, milking, and blood sampling.

A long-day photoperiod (LDPP) was used, with 16 h daylight and 8 h of dim night light (Fig 4). The cows were moved into the Light lab 22 days prior to the onset of the light experiment, to allow them to acclimatize to the surroundings. Thereafter the treatment period started and only LED lighting was employed for 33 days. During period 1, the daylight intensities were Blue_9_ and Red_9_, respectively. During period 2, White_9_ was used as the White light treatment, while for the White-Blue treatment, White_9_ was turned on for 10 h and switched to Blue_9_ for the last six hours of the daytime period. All daylight treatments had the same dim night light, White_1_ (Table 1). The light intensities selected during daytime was a result from experiment I combined with practicalities as ensuring a safe work environment for barn staff.

#### Standing and lying behavior

Standing and lying activity was recorded by HOBO Pendant G Data loggers, model UA-004-64 G (ONSET, Bourne, Massachusetts, USA), attached to one hind leg of each cow, throughout the experimental period. Loggers and data were handled using the protocol suggested by UBC AWP [28]. The logger was set to record position (standing or lying) every five minutes. Number of standing and lying observations were summarized first per day (24h), daytime (16h), and night-time (8h), and proportion of standing/lying time was calculated as number of standing/lying observations divided by total observations per day, daytime, or night-time. Number of standing and lying bouts, and bout durations, were also measured, according to standard operating procedures [28].

#### Feed intake

Silage provided *ad libitum* was replaced daily (0545h) and topped up twice daily (1300 and 1930h). Concentrate was fed four times per day (0545, 1300, 1630, and 1930h), on top of the silage. Every cow had their own feeding through providing individual feed intake. Daily concentrate ration was adjusted to the calculated requirements for individual milk yield according to the NorFor system [29]. Chemical composition of silage, based on samples from the silo and analyzed with near-infrared reflectance spectroscopy, and of concentrate, is shown in Table 2. Silage 1 was fed in period 1 and silage 2 in period 2. In both periods, cows were fed a mix of concentrate 1 and 2. The ratio of the two concentrates were adjusted to ensure an equal crude protein intake. Silage refusals were collected manually before morning and evening feeding for five consecutive days at the end of the treatment period, to ensure feed intake for daytime and night-time, respectively. There was concentrate in the silage refusals on very few occasions. The refusals were weighed, and silage ration was adjusted individually to ensure *ad libitum* feeding. Silage samples were taken from each feeding and stored in a plastic bag at -20° C until analyzed. Samples from two weeks were pooled and analyzed for dry matter (DM) content by first drying at 60°C overnight, grinding, and then drying at 60°C overnight [30]. Energy balance was calculated according to the NorFor system [29].

**Table 2:** Chemical composition of silage and concentrate.

Item	Silage 1	Silage 2	Concentrate 1	Concentrate 2
Period 1 (% of diet)	67	-	25	8
Period 2 (% of diet)	-	60	36	5
Dry matter (DM, g/kg)	391	464	880	890
Ash (g/kg DM)	76	114	-	-
Crude protein (g/kg DM)	139	181	170	280
Neutral detergent fiber (g/kg DM)	424	411	260	250
Metabolizable energy (MJ)	10.6	10.7	13.3	14

Both fed in Experiment II to lactating dairy cows exposed to light of different wavelengths during 33 days. Silage 1 was fed in period 1 and silage 2 in period 2 and a mix of concentrate 1 and 2 was fed in both periods to ensure an equal crude protein intake.

#### Milk

The cows were milked twice daily at 0615 and 1700h (DeLaval DelPro MU480), and milk yield was recorded automatically. Milk was sampled for five consecutive days at morning and evening milking at the end of the treatment period. Milk samples were obtained throughout milking with the Tru-Test technique (Tru-Test Mechanical Milk Meter (MM6) DeLaval AB, Tumba, Sweden), preserved with 10% bronopol, (2-bromo-2-nitropopane-1·3-diol VWR International AB, Stockholm, Sweden), stored at 8°C, and analyzed within five days.

Milk samples were individually analyzed for content of fat, protein, lactose, and somatic cell count, using infrared Fourier-transform spectroscopy (CombiScope FTIR 300 HP, Delta Instruments B.V., Drachten, The Netherlands). The mean value for 10 milk samples for each period were used in the statistical analyses. Energy-corrected milk (ECM) yield was calculated based on fat, protein, and lactose content according to Sjaunja *et al*. [31].

#### Melatonin and IGF-1

Blood was sampled from the tail vein (*v*. *caudalis mediana*) four times (at 0830, 1600, 2230 and 0400h) during the last 24 hours of the treatment period. The samples were collected in tubes containing Na-EDTA (0.9 x 38 mm; Vacutainer No. 360215; BD; Franklin Lakes, NJ) and placed on ice immediately after collection. Plasma aliquots were obtained after centrifugation for 10 min at 4000 x g and stored at -20°C until analysis, within one day of sampling. A commercial ELISA kit was used for analyzing melatonin (IBL International 2014) and IGF-1 (Mediagnost 2018). Average sensitivity and intraassay and inter-assay coefficient of variation was 0.09 μg/mL, 2.5%, and 7%, respectively, for IGF-1 (10 assays), and 1.6 pg/ml, 5.3%, and 15%, respectively, for melatonin (13 assays).

### Statistical analysis

The mixed procedure in SAS (SAS version 9.4, SAS Institute Inc., Cary, NC.) was used to test whether pupil size was affected by light color (blue, red or white) or light intensity (level 1-9/10). Color, intensity, and their two-way interaction were included as fixed effects, and cow nested within treatment as a random effect, with an unstructured covariance structure.

To test whether standing, lying, milk production, feed intake, melatonin, or IGF-1 was affected by light treatment (Blue, Red, White, or White-Blue), the mixed procedure in SAS was used. In all models, treatment and period (first or second) were included as fixed effects, and cow nested within treatment as a random effect, with an unstructured covariance structure. The model for standing and lying also included the fixed effect of time of day (day and night); the model for milk yield, milk composition, and feed intake included the fixed effect of days in milk; and the model for melatonin and IGF-1 included the fixed effect of sampling time (0830, 1600, 2230, 0400h). Interactions of fixed effects were excluded using stepwise backwards elimination; any interaction effect with P>0.10 was excluded from the model until all remaining interactions showed P<0.10. The two-way interaction of treatment × period was kept in the models for standing, lying, milk yield, milk composition, and feed intake, and the three-way interaction of treatment × period × sampling time was kept in the models for melatonin and IGF-1. Melatonin and IGF-1 were also tested for correlation, both within 24 hours and at the sampling times (0830, 1600, 2230, 0400h), using the correlation procedure in SAS.

Values presented are least squares mean (LSM) ± standard error of the mean (SEM), unless otherwise stated. Results were considered significant at P≤0.05, while a trend was assumed for probabilities 0.10 > P > 0.05. Post-hoc means separation for significant main effects was applied using Tukey-Kramer’s adjustment of probability values.

## Results

### Experiment I

**Fig 5:**
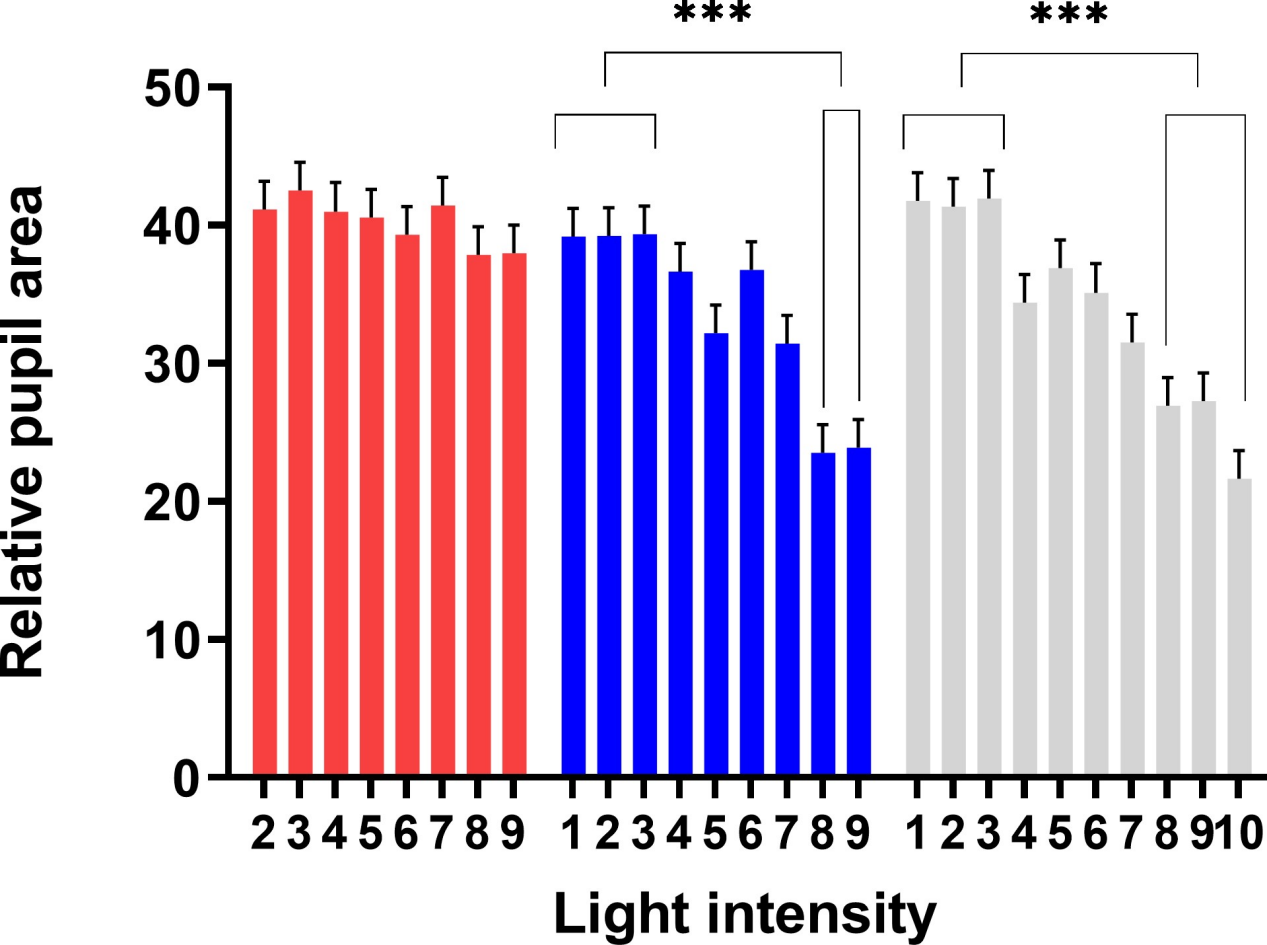
Relative area of the pupil (RAP) of cows exposed to Red, Blue, and White light intensity. There was no difference in relative pupil area for cows under red light. Under blue and white light, the relative pupil area decreased by almost half (***p <0.001).

There was no significant difference in RAP for the red light intensities tested in experiment I, despite an almost 100-fold increase in photon flux (Fig 5). The average RAP over the entire range of red light intensities tested was 40±1.2%.

**Fig 6:**
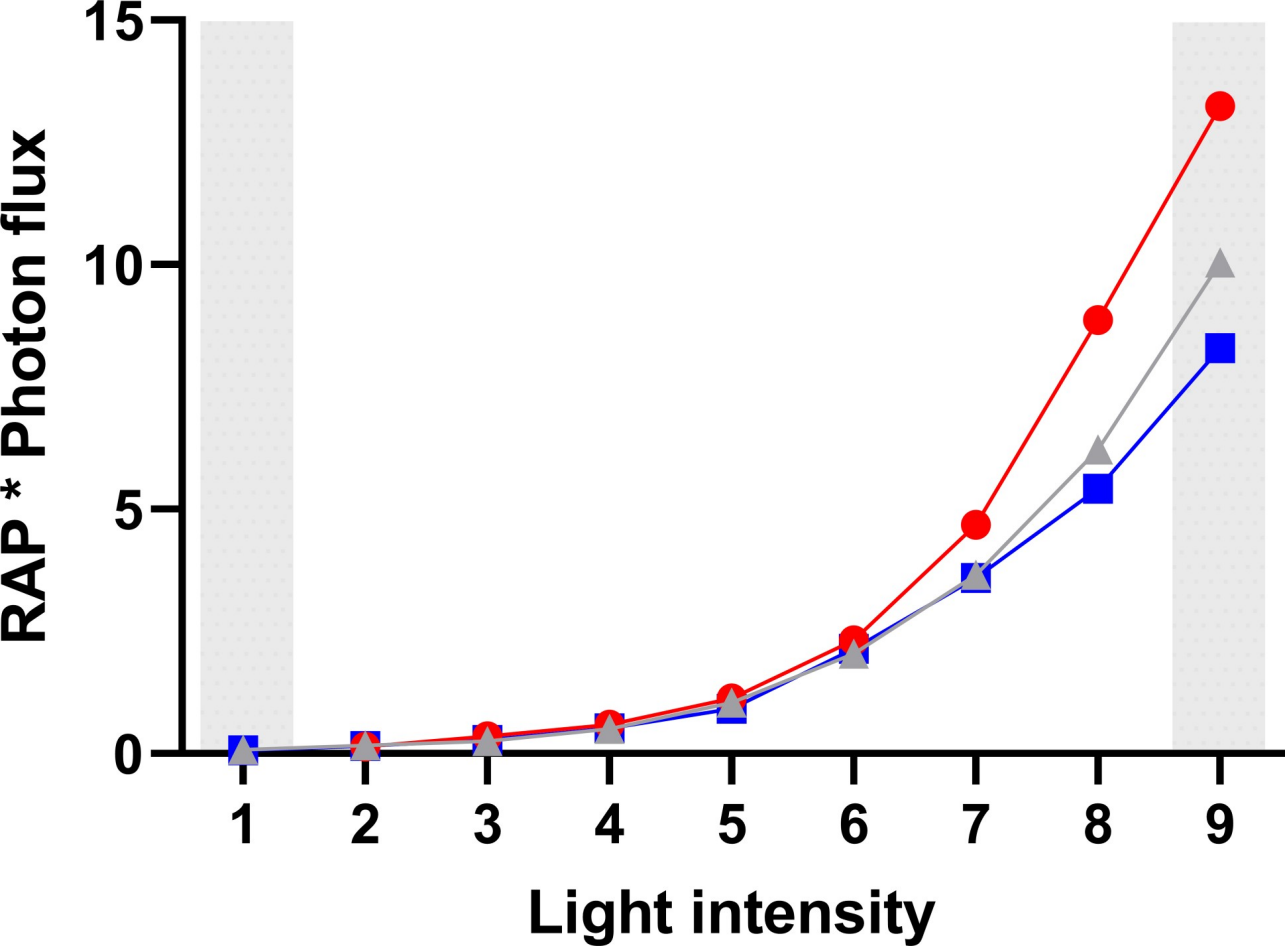
Relative number of photons reaching the retina when different light-emitting diode (LED) lights were employed. Grey area indicates dim night light treatment and daylight treatment in Experiment II.

In contrast, the brightest blue and white lights produced significant constriction of the pupils, whereas there were no differences for light intensities from 1 to 3 (p = 1) in experiment I. On increasing from Blue_3_ to Blue_8_, RAP decreased from 39.5±2.1% to 23.5±2.1% (p <0.001) and from 42±2.1% to 27±2.1% using white light (p <0.001). The average RAP for Blue_9_ was 24±2%, for Red_9_ 38±2%, and for White_9_ 27.5±2%. In dim night light (White_1_ used in all the daylight treatments in experiment II), the average RAP was 42±2%. The relative number of photons reaching the retina (RAP x photon flux) for Blue_9_ was 8.3, for Red_9_ 13.2, and for White_9_ 10.0. For the dim night light (White_1_), the relative number of photons reaching the retina was 0.08 (Fig 6), implying that the relative number of photons reaching the retina during daylight conditions was approximately 100 to 165 times higher than in dim night light.

### Experiment II

#### Standing and lying behavior

**Fig 7:**
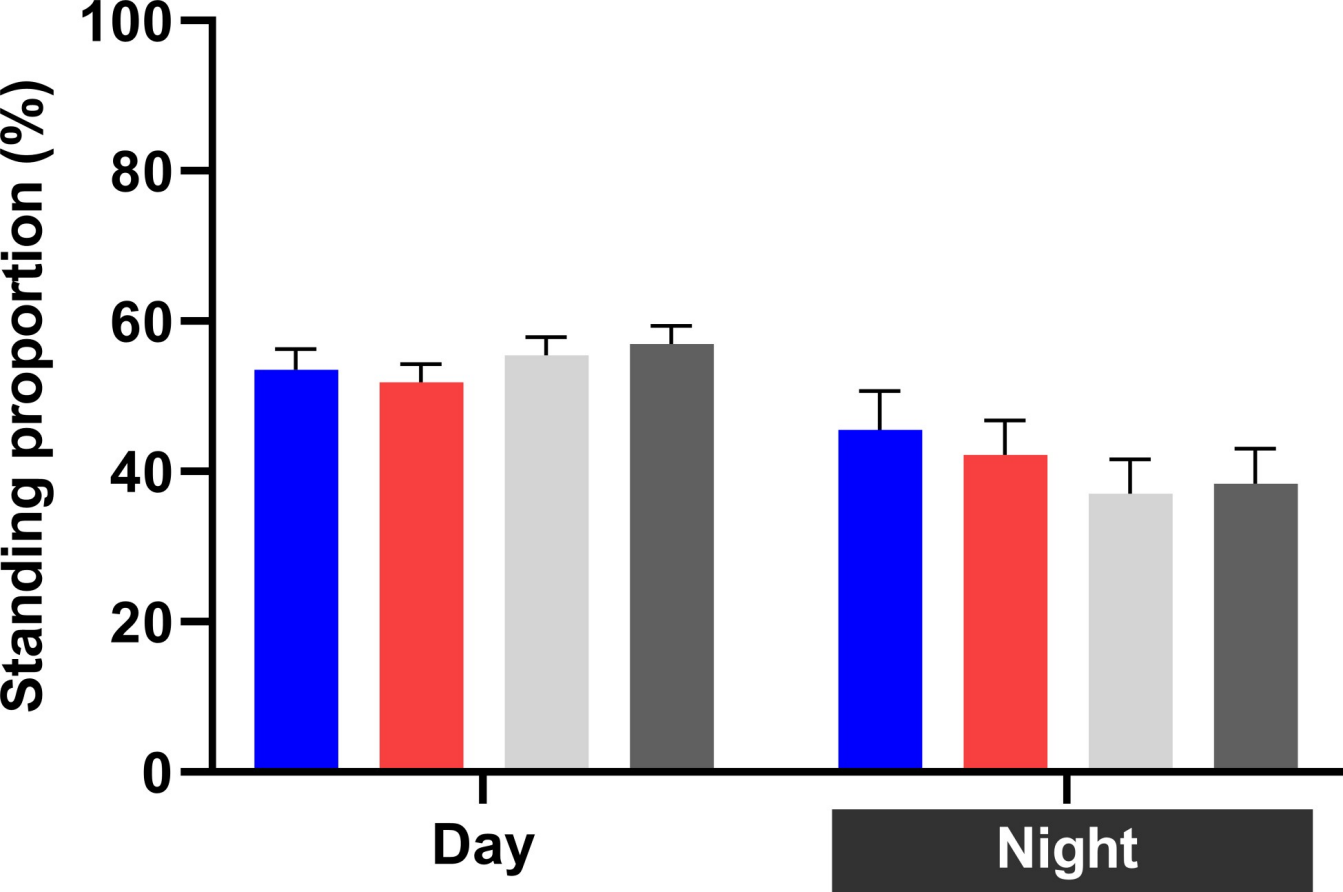
Treatment least squares means (LSM) for activity. Standing proportion per treatmeant, and in daytime (16 h) and night-time (8 h).

Light treatment did not affect cow activity, with an overall standing proportion of 54±1% during daytime and 40±2% during night-time (Fig 7). The overall number of standing bouts was 6±0.4 bouts during daytime and 3±0.2 bouts during night-time. Mean standing bout duration was 78±4 min during daytime and 41±3 min during night-time.

The overall mean number of lying bouts was 8±0.3 bouts during daytime and 4±0.2 bouts during night-time. Mean lying bout duration was 59±2 min during daytime and 55±3 min during night-time.

#### Feed intake and milk yield

There was no difference in feed DM intake (DMI) between the treatments (P>0.1) (Table 3). There was no difference in calculated energy balance between the treatments (p>0.7). Milk yield (kg) was maintained during the five weeks of treatments, with no difference between treatments (P = 0.1). Additionally, the treatments did not affect ECM (P = 0.3), fat content (P = 0.2), protein content (P = 0.4), or lactose content (P = 0.6).

**Table 3:** Least squares mean (LSM) ± standard error of: Milk yield, energy-corrected milk (ECM), milk composition, dry matter intake (DMI), and energy balance for cows exposed to the Blue, Red, White and White-Blue light treatments.

Variable	Blue	Red	White	White-Blue	P-value
Milk yield (kg)	32.3 ± 1.4	28.5 ± 1.4	32.2 ± 1.4	32.2 ± 1.4	0.16
ECM (kg)	33.2 ± 1.4	31.3 ± 1.3	33.7 ± 1.3	34.9 ± 1.4	0.32
Milk fat (%)	4.2 ± 0.2	4.7 ± 0.2	4.2 ± 0.2	4.6 ± 0.2	0.20
Milk crude protein (%)	3.8 ± 0.1	3.9 ± 0.1	3.8 ± 0.1	3.9 ± 0.1	0.42
Milk lactose (%)	4.4 ± 0.05	4.5 ± 0.05	4.4 ± 0.05	4.4 ± 0.05	0.61
DMI (kg)	24.8 ± 0.9	25.4 ± 0.9	25.4 ± 0.9	26.5 ± 0.9	0.57
Energy balance (%)	99.7 ± 2.6	97.4 ± 2.6	95.3 ± 2.6	95.5 ± 2.6	0.67

#### Melatonin and IGF-1

**Fig 8:**
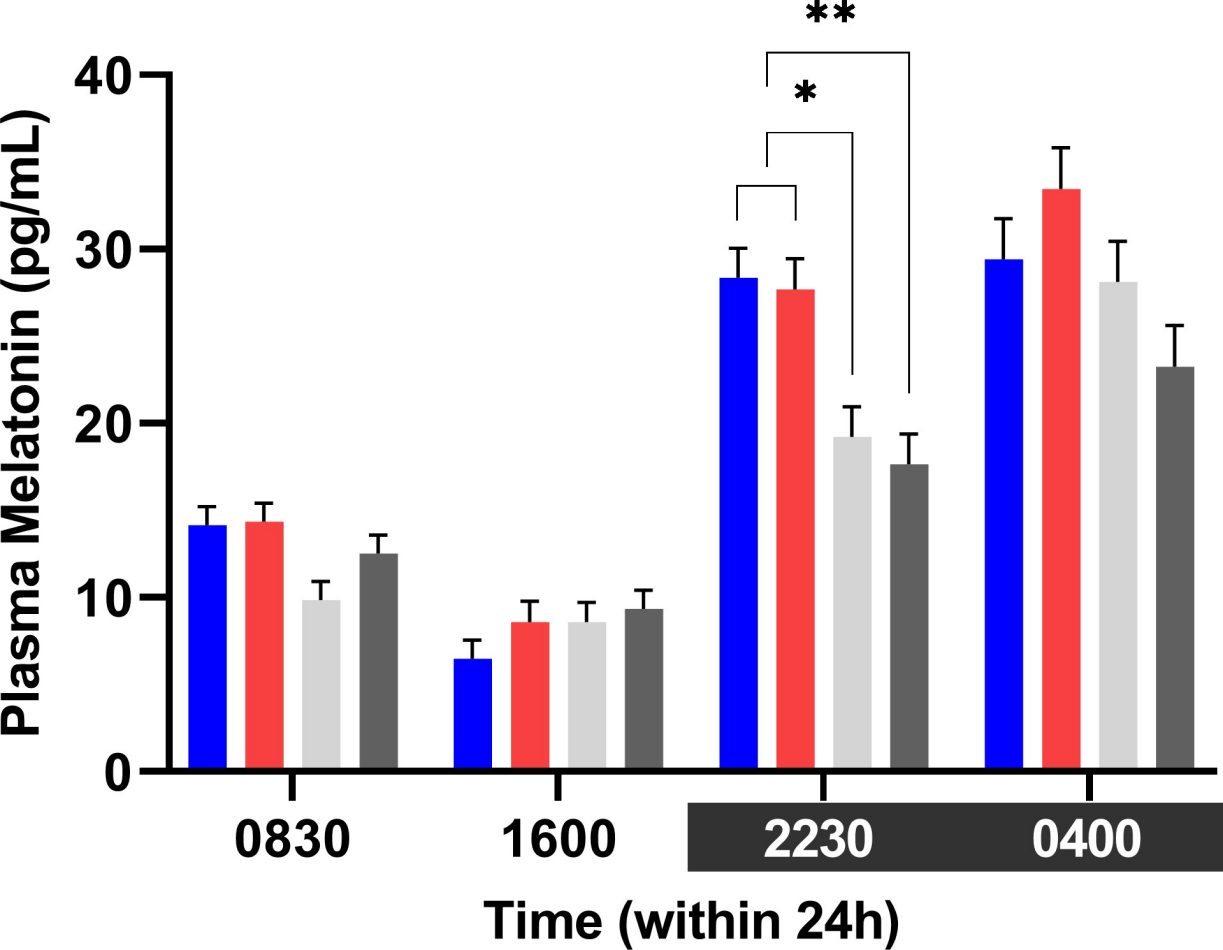
Treatment least squares mean (LSM) of plasma melatonin within 24 hours. Cows exposed to the Blue and Red light treatments had higher plasma melatonin than cows exposed to White light (*p <0.1) and White-blue light (** p<0.05).

Plasma melatonin was higher during dim night light than during daylight (P<0.001) (Fig 8). At 2230h, melatonin was significantly higher (p<0.05) for cows exposed to Blue or Red light during the day (27.7±1.7 pg/ml vs. 28.3±1.7 pg/ml) than cows exposed to White-Blue light (17.6±1.7 pg/ml), and tended to be higher (p<0.1) than in cows exposed to White light (19.2±1.7 pg/ml). The highest melatonin levels were detected at 0400h (P<0.001) in all treatments (28.6±1.2 pg/ml), when no difference was found between treatments (p>0.8). At 1600h, the lowest melatonin level (P<0.001) was detected in all treatments (8.2±0.5 pg/ml). No significant difference between the light treatments (p >0.8) was observed at 1600h or at 0830h.

**Fig 9:**
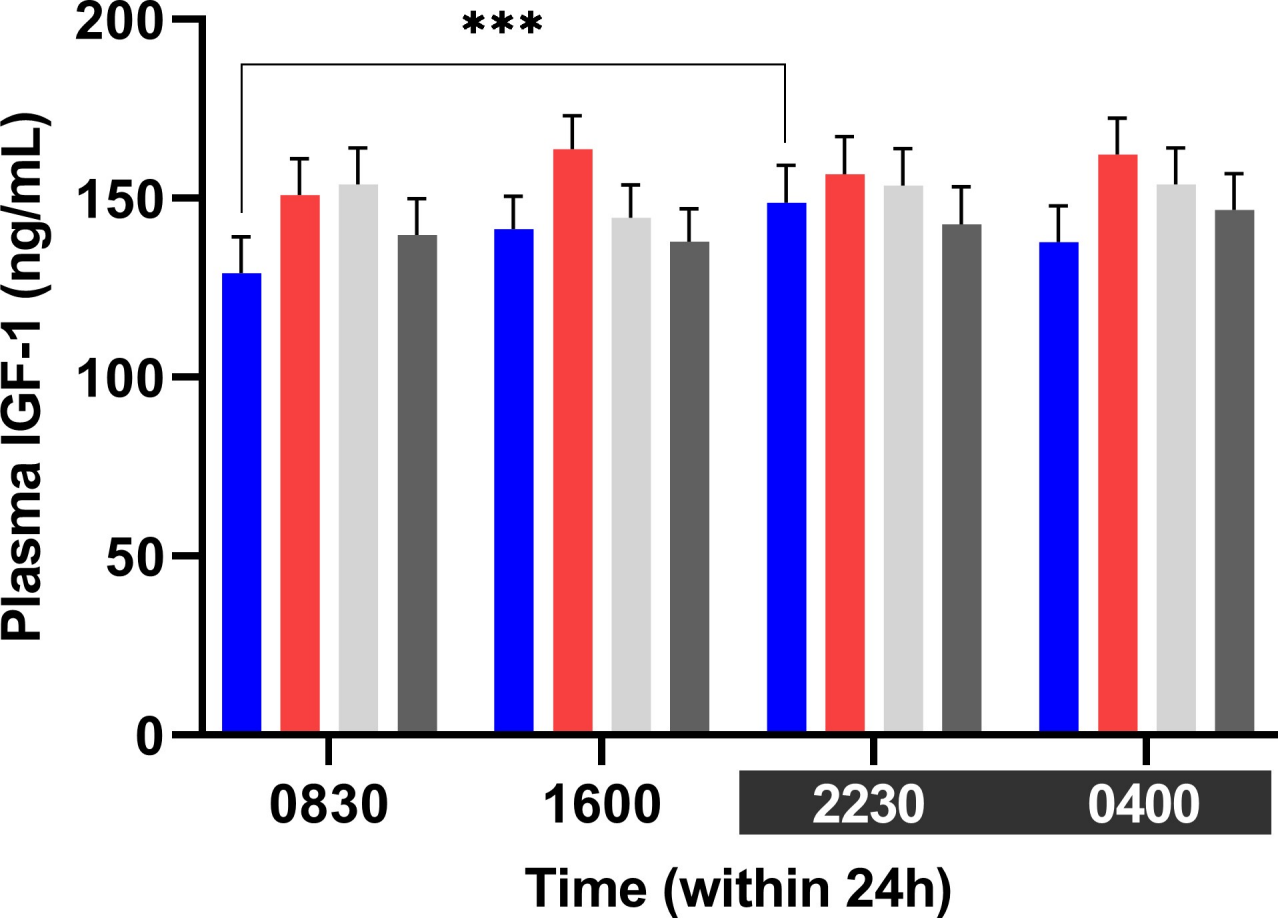
Treatment least squares mean (LSM) of plasma IGF-1 within 24 hours. Cows exposed to the Blue light treatment had higher plasma IGF-1 concentration at 2230 h than at 0830 h (*** p < 0.001).

Plasma IGF-1 concentration was higher at 2230 h than at 0830 h for cows in the Blue treatment (148.8±10.4 ng/mL vs. 129±10.2 ng/mL) p = 0.0002) (Fig 9). No difference was observed within the other treatments. Including all treatments, IGF-1 concentration was lowest (P<0.001) at 0830 h (143.4±5.1 ng/mL and highest (P<0.001) at 2230 h (150.5±5.2 ng/mL. No correlation was observed between melatonin and IGF-1 concentrations within 24 hours.

## Discussion

Red light, regardless of intensity, had no significant effect on pupil size, with the RAP value obtained under red light being similar to that obtained under the dim night light (White_1_) conditions. In contrast, bright blue and bright white lights constricted the pupils effectively, contradicting our hypothesis (i). This difference in efficacy between short and long light wavelengths is well-established in some other diurnal mammals, including humans [32]. Pupillary constriction is largely mediated through ipRGCs, but these light-sensitive ganglion cells also receive input from retinal cones and rods [13, 33]. However, cone inputs contribute less than a minute to pupillary constriction when steady-state levels of light are used, whereas rods may contribute longer, but only at light levels below saturation of the rod response [24]. In our experiments, steady levels of light were maintained for several minutes, implying no or very little cone input. Additionally, most of the light intensities tested were clearly above, the mesopic range of the bovine retina, suggesting that rod input was low. Peak absorption of melanopsin has been shown to be approximately 480 nm in other species [34–36]. The blue and white lights used in our study (the latter containing a substantial amount of short to medium wavelengths) were therefore strong stimuli for melanopsin-based photoreception, whereas red light was barely absorbed by melanopsin. Under low light intensities, our results showed no difference in pupillary size between the light colors’, which indicates that our results on pupillary constriction were not affected by environmental factors, e.g., stress [37]. This led us to conclude that the pupillary responses to the different lights in our experiments were mainly melanopsin-driven.

The longer resting time observed during the dark hours corresponds with results reported by Suarez-Trujillo *et al*. [38]. The lack of differences in activity between treatments may be an effect of the tie-stall system used, which restricts activity per se compared with loose housing. We did not detect any specific patterns in the activity data, e.g., whether all cows were standing or lying down at the same time, contradicting our hypothesis (ii). However, changes in activity as a result of spectrally different lighting regimes may be more apparent in a loose housing system, an issue which warrants further investigation.

The diurnal rhythm in melatonin concentrations observed here, with the highest concentrations during the dark period, confirms previous findings in dairy cows [38–40] and younger cattle [22, 41–45]. The melatonin concentration increased rapidly on switching to a low light intensity, which is consistent with previous results [22, 42]. The peak melatonin level was found in the second set of samples after onset of darkness (after 7 hours in the dark), confirming results in several other studies [41, 43, 45, 46]. Interestingly, the Red and Blue light treatments caused a more rapid increase in melatonin after the onset of darkness than the White and White-Blue light treatments, contradicting our hypothesis (iii). This could be a period-treatment confounding effect, or cessation of the intense red and blue lights may have elicited more rapid secretion of melatonin. The White-Blue treatment (White_9_ light for 10 h, Blue_9_ light for 6 h) did not cause such a rapid increase in melatonin at night as seen with the Blue treatment (Blue_9_ light for 16 h). Thus, the shorter exposure to blue light before the dim night light in the White-Blue treatment may not have been sufficient for a rapid response in melatonin secretion. The highest melatonin levels were obtained after the long-wavelength Red daylight treatment, although the levels were not significantly different from those in the Blue and White treatments. Elsabagh *et al*. [22] found that two hours of dim yellow LED light increased melatonin concentration faster than two hours of dim short-wave blue LED light treatment, which suggests that dimming light after exposure to longer wavelengths is at least as effective in replenishing plasma melatonin levels as when short-wavelength light has been employed during the day. However, the light intensities used by Elsabagh *et al*. [22] were similar to our light intensity 2 and only 8-week-old calves were studied, which makes comparison with our results more difficult.

Throughout the study period in experiment II, DMI and milk yield were maintained in all treatments. Although, milk yield can be expected to decline post peak lactation [47]. Since both DMI and milk yield were maintained it suggests that LED light regardless of color stimulated a more persistent lactation. However, earlier studies have showed that a LDPP increased milk yield when compared to NDPP [3]. In our study, the maintained milk yield might be an effect of the LDPP, the effect could also be a result of the maintained DMI and the positive energy balance. Despite no effect of treatments on DMI, actual nutrient intake is unknown and may have been moderately affected by actual intake proportions of forages and concentrates. However, daily visual inspection indicated that concentrate intake was complete, and thus, confounding from this factor is unlikely. To give the concentrate in a separate bowl might be preferable, though it was not manageable in this barn due to the construction of the head fronts. In addition, when the cows moved into the Light lab, there was a change in both their environment and their milking system, from an automatic quarter milking system to a cluster milking system. A Light lab with the automatic quarter milking system used in standard management of cows in the herd could have helped to study the impact of the lighting conditions alone. No effects on milk composition caused by the prolonged photoperiod were observed, which is in agreement with previous studies [3, 46].

Cows in the Blue treatment in experiment II showed a tendency for a diurnal pattern in plasma IGF-1, though none of the other treatments indicated a diurnal pattern in IGF-1. This corresponds with earlier findings in one study [44] but not in others [3, 48]. Our results showed no correlation between plasma levels of melatonin and IGF-1 throughout the 24 hours when samples were taken. Muthuramalingam *et al*. [44] discovered a tendency for a negative correlation between IGF-1 and melatonin during night-time. However, other factors not measured in the present study may also have caused variation in circulating IGF-1, and some of these factors may have influenced plasma levels more than the light treatment. Negative energy balance is one factor that causes a decrease in circulating IGF-1 [49, 50], and often arises close to the onset of lactation [51]. Negative energy balance can explain findings that the number of days in milk, counted from the onset of lactation, and IGF-1 are positively correlated [52, 53]. In addition, IGF-1 plays an important role during pregnancy, in gonadotropin-induced folliculogenesis [50], meaning that days in pregnancy can affect the dynamics of circulating IGF-1. All cows in experiment II were pregnant, within the range of post peak lactation and prior to month 7 of pregnancy. In a previous study on cows treated with LDPP, Dahl *et al*. [3] observed increased concentrations of IGF-1 that were independent of changes in growth hormone and IGF-binding-proteins-2 and -3. A later study by Kendall *et al*. [48] showed increased concentrations of IGF-1 in LDPP calves, regardless of nutritional status. The IGF-1 concentrations reported in the literature differ markedly [3, 44, 48, 49, 52, 53], possibly due to the factors mentioned above and/or the method of analysis used in the laboratory. Our plasma IGF-1 results are similar to those obtained in a pilot study performed by Ferneborg *et al*. [54] on the same herd and with the same method of analysis. However, the number of animals in the present study was insufficient to give the statistical power needed to detect differences below 25 ng/ml.

It is interesting that melatonin and activity levels, two parameters related to diurnal rhythm, were essentially similar regardless of daylight regime. The light-driven circadian oscillator (process C) is required for partitioning sleep during the day-night cycle, whereas prolonged periods of wakefulness increase the propensity to sleep (homeostatic mechanism or process S) (see Borbely *et al*. [55] for review). In experiment II, we used a period of acclimatization before feed and milk data were sampled. Sampling for melatonin and IGF-1 analyses, and activity measurements, were made at the end of each trial period (lighting regime). Hence, we believe that our data mainly reflect the effect of the different daylight regimes on the light-driven circadian oscillator.

Both short and medium wavelengths, which are easily absorbed by ipRGCs and short-wavelength (blue) cones, had a similar effect to long-wavelength (red) light, although red light is unlikely to be absorbed by melanopsin, at least to any substantial degree. We do not believe that the higher RAP we observed for Red daylight could compensate for the poor absorption by ipRGCs. It has been shown in transgenic mice that both the rod-cone and melanopsin-driven pathways are required for normal entrainment of the circadian rhythm, and thereby the sleep cycle [8]. Therefore, it is more likely that the sleep cycle in cows under red light conditions is driven by medium- to long-wavelength cone input to ipRGCs, whereas blue and white daylight can affect both cone types in the bovine retina, as well as the melanopsin-pathway directly. Thus, we suggest that the retinal circuitry conveying light signals to the circadian oscillator in the cow shares basic features with that of both mouse and human.

In conclusion, our results show that LED fixtures emitting red light driving the ipRGCs indirectly via ML-cones, blue light stimulating both S-cones and ipRGCs directly and a mixture of wavelengths (white light) exert similar effects on milk yield and activity in dairy cows. Furthermore, the feed intake required is not significantly different between light treatments. This suggests that the spectral composition of LED lighting in a barn is secondary to duration and intensity. Thus, the choice of spectral composition better be based on other preferences, such as visual comfort for barn staff and suitable lighting for surveillance systems. However, long-term effects of LED lighting with different spectral compositions on production parameters, as well as activity and sleep patterns in dairy cows in loose housing, warrant further investigation.
